# Neurobehavioral Signatures of Epileptogenesis: Molecular Programs, Trait-like Phenotypes, and Translational Biomarkers Beyond Seizures

**DOI:** 10.3390/ijms27052511

**Published:** 2026-03-09

**Authors:** Ekaterina Andreevna Narodova

**Affiliations:** Department of Neurology, Prof. V.F. Voyno-Yasenetsky Krasnoyarsk State Medical University, 660022 Krasnoyarsk, Russia; katya_n2001@mail.ru

**Keywords:** epileptogenesis, biomarkers, synaptic plasticity, neuroinflammation, neurobehavioral phenotypes, disease modification

## Abstract

Epileptogenesis is commonly defined by the emergence of spontaneous seizures after an initial insult; however, convergent experimental and clinical evidence indicates that the underlying disease process begins well before seizures become clinically detectable. During this pre-seizure phase, persistent molecular cascades remodel synaptic plasticity, circuit architecture, and glial–immune signaling. These processes are associated with trait-like alterations in cognition, affect, and behavior. Despite their clinical relevance, these neurobehavioral signatures remain poorly integrated into molecular models of epileptogenesis and are rarely considered as translational biomarkers of disease progression. This review synthesizes evidence linking core epileptogenic molecular cascades—maladaptive synaptic plasticity, glial–immune signaling, oxidative–metabolic stress, and activity-dependent gene regulation—to reproducible alterations in executive control, cognitive flexibility, emotional regulation, and motivational–social behavior. We outline an integrative framework in which these phenotypes are conceptualized as system-level readouts of progressive network reconfiguration rather than nonspecific “comorbidities” or mere consequences of recurrent seizures. Within this perspective, neurobehavioral markers can complement electrophysiological and molecular measures by capturing disease-relevant changes during windows when anti-epileptogenic interventions would be most effective. To increase mechanistic specificity, we provide representative pathway and gene-level anchors across epileptogenesis stages, a structured molecular-to-neurobehavioral mapping, and an operational biomarker panel specifying confounders and minimal controls. These anchors are included to ground the framework in experimentally documented molecular nodes with stage-dependent relevance; examples are representative rather than exhaustive, and evidence strength is indicated as preclinical mechanistic versus associative human observations. Finally, we discuss methodological requirements for biomarker validity (specificity, temporal anchoring, and cross-model consistency) and outline how integrating molecular and neurobehavioral trajectories may refine target discovery and improve the translation of anti-epileptogenic strategies. Conceptualizing epileptogenesis as a progressive disease process with measurable pre-seizure neurobehavioral signatures may broaden biomarker strategies beyond seizure occurrence and support the development of disease-modifying interventions.

## 1. Introduction

Epileptogenesis is increasingly conceptualized as a progressive disease process rather than a discrete transition from a “normal brain” to recurrent seizures. After an initiating insult—such as status epilepticus, traumatic brain injury, stroke, infection, or a genetically mediated developmental disturbance—molecular and cellular cascades remodel synaptic function, circuit architecture, and glial–immune signaling over weeks to months. This interval can be clinically deceptive: seizures may be absent, infrequent, or insufficient to reflect ongoing network reconfiguration. The central challenge is therefore twofold: to identify markers that track epileptogenic progression during pre-seizure or early-course windows and to translate these markers into interventions that modify disease course rather than solely suppress symptomatic events [1,2,3,4].

Most biomarker efforts in epilepsy have focused on electrophysiological, imaging, and molecular readouts—including interictal epileptiform activity, network connectivity patterns, neuroinflammatory mediators, transcriptomic signatures, and proteomic candidates. In parallel, the broad experimental and clinical literature indicates that stable changes in executive control, cognitive flexibility, affect regulation, and motivational–social behavior can accompany epileptogenic remodeling. Yet these neurobehavioral alterations are often categorized as nonspecific “comorbidities” or attributed to recurrent seizures, medication effects, or psychosocial burden. These explanations are often plausible but do not fully account for early, seizure-independent network remodeling. Convergent experimental and clinical findings suggest that behavioral and cognitive changes can emerge early—sometimes before established epilepsy—and can track underlying circuit reorganization in models where seizure exposure is experimentally controlled [5,6,7,8].

In this review, trait-like neurobehavioral signatures are examined as system-level readouts of progressive network reconfiguration and as candidate translational biomarkers of epileptogenesis. The term “signature” is used here deliberately to emphasize reproducible patterns rather than single symptoms. Neurobehavioral measures integrate distributed molecular and circuit processes into repeatedly assessable functional outcomes that may be sensitive to early disease progression and are clinically interpretable. If validated with appropriate methodological safeguards, such signatures could complement molecular and electrophysiological markers, improve temporal anchoring of disease stage, and refine outcome selection for trials of anti-epileptogenic strategies [9,10,11,12].

The goal is not to replace molecular biomarkers with behavioral measures, but to integrate them within a multiscale framework. Epileptogenesis involves molecular cascades, circuit reorganization, and altered information processing; neurobehavioral measures capture the organism-level functional consequences of these multiscale changes. This review integrates defined molecular cascades, reproducible neurobehavioral domains, cross-level mechanistic mapping, and methodological criteria for biomarker validity.

Although neurobehavioral signatures are discussed at the organism level, the present framework is explicitly grounded in molecular neurobiology, with behavioral phenotypes treated as downstream functional readouts of molecular and circuit-level epileptogenic programs [13,14,15,16].

Importantly, this framework is grounded in convergent molecular evidence across synaptic, inflammatory, metabolic, and gene regulatory pathways implicated in experimental and clinical epileptogenesis. Because this is a narrative, biomarker-oriented review, we provide representative pathway- and gene/node-level anchors and explicitly indicate evidence strength, rather than attempting exhaustive quantitative effect-size synthesis.

### Scope, Definitions, and What This Review Adds

In this review, epileptogenesis denotes the progressive transformation of brain networks that increases the propensity for spontaneous seizures, encompassing molecular, cellular, and circuit-level changes that precede, accompany, or follow the first seizures. We use the term “molecular programs” as an operational shorthand for recurrent, mechanistically defined molecular modules (i.e., sets of signaling pathways, transcriptional/epigenetic regulators, and effector genes/proteins) that are repeatedly implicated across epileptogenesis models and that exert convergent effects on synaptic function, glial–immune coupling, cellular metabolism, and network excitability. The term “molecular programs” denotes experimentally documented and mechanistically defined molecular modules with identifiable pathways and measurable nodes. Where possible, we explicitly name representative pathways and molecular nodes within each module (e.g., IL-1R/TLR4–NF-κB signaling; TNF-α/TNFR1; mechanistic target of rapamycin (mTOR); brain-derived neurotrophic factor (BDNF)–TrkB signaling; Nrf2 antioxidant response; DNA methylation/HDAC-dependent chromatin regulation; selected microRNAs). Examples are representative rather than exhaustive, and causal evidence varies by model and stage.

The scope includes post-injury and genetic or developmental pathways to epilepsy, with emphasis on mechanistic convergence rather than exhaustive etiological cataloging. Because the clinical concept of a “pre-seizure” window is context-dependent, we use it pragmatically to refer to stages in which epileptogenic remodeling is underway but recurrent spontaneous seizures are not yet established or are not sufficient to represent stable epilepsy [4,17,18,19].

For the purposes of this review, epileptogenesis is defined as a stage-dependent trajectory comprising three partially overlapping phases: (i) pre-seizure molecular and cellular remodeling initiated by an insult and characterized by activity-dependent transcription, synaptic reweighting, and glial–immune engagement; (ii) emergence of early network hyperexcitability and reduced resilience, reflected in altered excitation–inhibition balance and unstable state transitions; (iii) transition to spontaneous recurrent seizures (SRS), representing a late and discontinuous clinical manifestation of preceding network reconfiguration.

Throughout this review, we explicitly distinguish compensatory or homeostatic plasticity—adaptive mechanisms that transiently stabilize network activity—from maladaptive plasticity and metaplastic bias that consolidate pathological operating regimes. Compensatory responses may initially buffer instability but can later fail, overshoot, or become incorporated into epileptogenic progression.

Neurobehavioral signatures are defined as reproducible, trait-like alterations in cognition, affect, or behavior that (a) emerge during epileptogenic remodeling, (b) map onto measurable molecular or circuit mechanisms, (c) are temporally anchored to pre-seizure or early disease stages. “Trait-like” does not imply immutability. It denotes stability relative to momentary state fluctuations and sufficient consistency to support longitudinal tracking. The domains emphasized here—executive control, cognitive flexibility, emotional regulation, and motivational–social behavior—were selected because they recur across models and clinical observations and plausibly index network functions vulnerable during epileptogenic reconfiguration [20,21,22,23]. Operationally, a “neurobehavioral signature” in the present framework is a reproducible pattern across task-defined readouts (rather than a single symptom), quantified by domain-relevant metrics (e.g., set-shifting/reversal learning errors and perseveration indices for cognitive flexibility; interference scores and response inhibition metrics for executive control; extinction learning rates or threat-bias indices for threat processing; effort discounting and progressive-ratio breakpoints for motivation). A phenotype is considered signature-like only if it (i) shows acceptable test–retest properties for longitudinal use, (ii) demonstrates temporal anchoring to pre-seizure/early-course stages, (iii) can be triangulated with at least one mechanistically relevant molecular, electrophysiological, or imaging marker.

This review provides a framework for aligning molecular epileptogenic cascades with neurobehavioral phenotypes under explicit biomarker-validity constraints. Instead of treating behavioral changes as “comorbidities” by default, we propose criteria under which they can be interpreted as system-level readouts of disease progression. We also delineate practical design principles for translational phenotyping—including confounding control, longitudinal anchoring, and cross-model triangulation—that can make neurobehavioral signatures informative endpoints for early-stage intervention studies.

In biomarker terms, the intended use of “neurobehavioral signatures” in this review is not diagnostic classification but (i) progression tracking during pre-seizure/early-course windows, (ii) stratification of vulnerability profiles, (iii) pharmacodynamic readouts in mechanistic early-phase studies. Accordingly, we treat a “signature” as a reproducible pattern across task-defined metrics with explicit temporal anchoring, confounder control, and triangulation with molecular, electrophysiological, and imaging markers. This stage-based definition is used throughout the review to organize molecular anchors, circuit-level changes, and neurobehavioral signatures within a temporally structured framework.

## 2. Materials and Methods

### 2.1. Literature Search Strategy

A structured literature search was conducted to identify experimental and clinical evidence on neurobehavioral alterations during epileptogenesis and their mechanistic links to molecular and circuit-level processes. Searches were performed in PubMed/MEDLINE, Scopus, and Web of Science. The initial search covered 01 January 2023 to 31 December 2025 and was updated on 30 January 2026. The search focused on English-language records published between 2009 and 2025.

Core search concepts combined epileptogenesis- and epilepsy-related keywords (e.g., epileptogenesis, antiepileptogenic, disease modification, post-injury epilepsy, latent period) with neurobehavioral keywords (e.g., cognition, executive function, cognitive flexibility, affect regulation, motivation, social behavior) and mechanistic keywords (e.g., synaptic plasticity, neuroinflammation, glial signaling, oxidative stress, metabolism, gene regulation, epigenetic remodeling). Boolean operators (AND/OR) and database-specific field tags were applied to refine queries. Representative full search strings for each database are provided in Table S1.

In addition to database searching, reference lists of key reviews and seminal experimental papers were manually screened to identify relevant publications not captured by the initial queries. Records were exported to a spreadsheet, and duplicates were removed prior to screening. The study selection process is summarized in a PRISMA-style flow diagram (Figure S1).

### 2.2. Eligibility Criteria and Synthesis Approach

Eligible sources included: (i) experimental animal studies of epileptogenesis with longitudinal or pre-seizure behavioral phenotyping; (ii) human observational or clinical studies reporting early-course or pre-seizure neurobehavioral alterations; (iii) translational studies linking behavioral phenotypes to molecular, electrophysiological, or imaging markers; (iv) mechanistic reviews addressing synaptic plasticity, glial–immune signaling, oxidative–metabolic stress, and activity-dependent gene regulation.

Studies focusing exclusively on seizure semiology or classification, surgical outcomes, or descriptive epidemiology without mechanistic relevance to epileptogenesis or without interpretable neurobehavioral endpoints were excluded. When multiple sources addressed overlapping concepts or mechanisms, more recent and/or mechanistically detailed publications were preferentially retained.

Screening and study selection were performed by the author using predefined relevance criteria applied consistently across databases. No formal risk-of-bias assessment or meta-analytic procedures were undertaken, given the focus on mechanistic and translational integration across heterogeneous experimental models and outcome measures. A quantitative meta-analysis was not undertaken due to substantial heterogeneity across experimental models, behavioral paradigms, molecular endpoints, and temporal staging.

### 2.3. Data Extraction and Conceptual Mapping

For each included publication, the following variables were extracted: study type (animal or human), epileptogenesis context (model or etiology; pre-seizure vs. early epilepsy), neurobehavioral domain assessed (executive control, cognitive flexibility, emotional regulation/threat processing, motivational–social behavior), molecular or cellular domain implicated (maladaptive synaptic plasticity, glial–immune signaling, oxidative–metabolic stress, activity-dependent gene regulation/epigenetics), and the level of mechanistic linkage provided (molecular, circuit, electrophysiological, imaging, or biomarker correlation).

**Figure 1:**
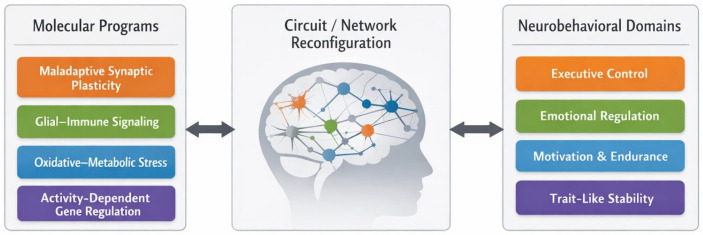
Multiscale framework linking molecular epileptogenesis to neurobehavioral signatures.

After deduplication, 570 records were screened and 170 full texts were assessed. Of these, 130 publications met predefined criteria for core qualitative synthesis. Additional methodological and conceptual references were incorporated to support the integrative framework, resulting in a final reference list of 166 sources. The range in retained records reflects inclusion of core studies for qualitative synthesis alongside additional conceptual/methodological sources supporting the framework. Extracted findings were organized to support the integrative mapping presented in Figure 1 and Table 1 and to derive biomarker-oriented design principles summarized in Section 6. The extracted domains and the conceptual mapping framework used for qualitative synthesis are summarized in Table S2. The final reference list therefore includes both core studies retained for qualitative synthesis and additional conceptual, methodological, and translational sources required to support the integrative framework. By integrating molecular epileptogenic programs with neurobehavioral phenotypes under explicit biomarker-validity constraints, this review provides a structured translational mapping rather than a purely descriptive synthesis.

## 3. Conceptual Framework: From Molecular Programs to System-Level Phenotypes

Epileptogenesis unfolds through coordinated adaptations across neurons, glia, and neurovascular–immune interfaces. At the molecular level, these adaptations include activity-dependent transcription, altered receptor trafficking, and shifts in excitation–inhibition balance. They also include neuroinflammatory signaling and metabolic/redox dysregulation.

These processes do not operate in isolation: they interact over time to reshape synaptic plasticity rules, circuit architecture, and network stability. Beyond increasing seizure susceptibility, these changes reduce network resilience, defined here as the capacity of neural systems to maintain stable and flexible function under stress [24,25,26,27].

The multiscale relationship between molecular processes, circuit reconfiguration, and neurobehavioral output is illustrated in Figure 1.

**Figure 2:**
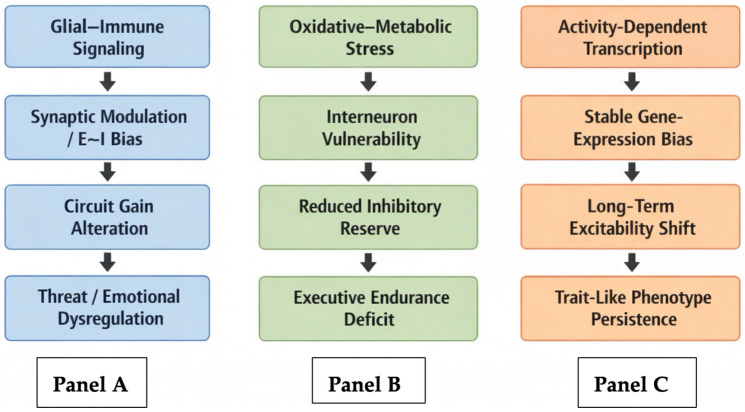
Mechanistic bridge cascades linking molecular domains to circuit and neurobehavioral phenotypes.

To further clarify mechanistic bridges between molecular domains and system-level phenotypes, a cascade-oriented schematic is provided in Figure 2.

(Panel A) Glial–immune cascade: Pro-inflammatory cytokine signaling (e.g., IL-1β, TNF-α) and complement-mediated synaptic remodeling alter excitatory–inhibitory balance and synaptic scaling within prefrontal–limbic and prefrontal–striatal circuits. These synaptic perturbations bias network gain and salience attribution, contributing to stable alterations in threat processing and emotional regulation.

(Panel B) Oxidative–metabolic cascade: Mitochondrial dysfunction and redox imbalance reduce inhibitory reserve and increase vulnerability of high-demand interneuron populations. Reduced inhibitory buffering destabilizes network gain control in executive circuits, leading to impaired executive endurance, reduced cognitive flexibility, and increased susceptibility to stress-related performance decline.

(Panel C) Activity-dependent transcriptional/epigenetic cascade: Sustained activity-dependent gene expression involving immediate early genes (e.g., FOS, ARC, EGR1), plasticity-related signaling pathways such as BDNF–TrkB and mTOR, epigenetic regulators including DNA methyltransferases (DNMT1/3A) and histone deacetylases (HDAC2/4), and regulatory microRNAs (e.g., miR-134, miR-146a) stabilizes altered synaptic and glial states. Through these mechanisms, transcriptional and epigenetic programs reshape synaptic architecture, receptor trafficking, and long-term circuit excitability, thereby contributing to persistent neurobehavioral phenotypes. This consolidation reshapes long-term circuit excitability and plasticity thresholds, providing a molecular substrate for trait-like neurobehavioral phenotypes across executive, affective, and motivational domains. Arrows represent preferential mechanistic progression (molecular → synaptic → circuit → behavioral level) and do not imply deterministic one-to-one causality.

Core molecular cascades implicated in epileptogenesis—maladaptive synaptic plasticity, glial–immune signaling, oxidative–metabolic stress, and activity-dependent gene regulation—interact to drive circuit and network reconfiguration. These changes manifest as stable alterations across executive, affective, and motivational domains. Seizures represent a late and discontinuous manifestation of a broader disease trajectory. Long before spontaneous seizures become frequent or clinically salient, networks may enter maladaptive operating regimes characterized by impaired inhibitory control, altered gain modulation, and reduced flexibility of network state transitions. Stable neurobehavioral changes may reflect underlying network reconfiguration even in the absence of overt seizures. Thus, neurobehavioral phenotypes should be viewed not solely as downstream consequences of epilepsy, but as potential indicators of the same network reconfiguration processes that ultimately support seizure generation [5,24,25,28].

Epileptogenesis is conceptualized here as a trajectory rather than a discrete threshold event. Molecular cascades alter synaptic and cellular function, biasing circuit dynamics and leading to measurable changes in behavior. Because neurobehavioral measures integrate distributed processes, multimodal validation is required for mechanistic interpretation [16,29,30,31]. In this review, neurobehavioral signatures are not assumed to be uniform across etiologies or experimental models. Instead, they are expected to cluster around vulnerable network functions—such as executive control, cognitive flexibility, emotional regulation, and motivation—that rely on distributed cortico-limbic and cortico-striatal circuits. These circuits are repeatedly implicated in epileptogenic remodeling through convergent molecular mechanisms, including synaptic plasticity alterations, glial–immune signaling, and metabolic stress. Rather than defining a single “behavioral phenotype of epileptogenesis,” this framework identifies families of signatures mapping onto specific molecular–circuit pathways and disease stages [32,33,34,35].

### Why Neurobehavioral Signatures Can Be Informative Biomarkers

Neurobehavioral measures offer several advantages within translational biomarker strategies for epileptogenesis. First, they can be assessed repeatedly over time, enabling longitudinal tracking of disease trajectories within individuals. Second, they are directly linked to network-level function and therefore may capture integrative consequences of distributed molecular and cellular changes. Third, they are inherently relevant to clinical outcomes, as alterations in cognition, affect, and motivation often represent meaningful sources of disability independent of seizure frequency. These features make neurobehavioral signatures attractive complements to electrophysiological and molecular biomarkers, particularly during pre-seizure or early stages of disease [36,37,38,39]. To avoid an undifferentiated construct, we distinguish domain-specific readouts, measurement properties required for longitudinal use, and mechanistic interpretability through multimodal triangulation. The validity of neurobehavioral signatures as biomarkers depends on three criteria: specificity to epileptogenic mechanisms, temporal anchoring to defined disease stages, and cross-model consistency across experimental and clinical contexts [9,10,11,40]. An additional consideration is scale alignment. Molecular biomarkers provide high specificity but limited integration, whereas neurobehavioral measures provide integrative functional readouts with lower mechanistic resolution. Multimodal strategies align these scales to improve interpretability. Within such an integrated framework, neurobehavioral phenotypes may be particularly useful for identifying windows of heightened plasticity or vulnerability—periods during which interventions targeting synaptic, inflammatory, or metabolic pathways are most likely to alter disease trajectory [15,19,24,25].

Within this framework, neurobehavioral signatures may inform target selection, endpoint definition, and timing of anti-epileptogenic interventions. When interpreted alongside molecular and electrophysiological markers, changes in these measures can provide early evidence of target engagement without relying solely on seizure frequency [11,19,40,41].

## 4. Molecular Cascades Implicated in Epileptogenesis

Epileptogenesis involves interacting molecular cascades rather than a single pathway, leading to changes in synaptic function, cellular homeostasis, and network dynamics. Across experimental models and clinical observations, four domains recur with notable consistency: maladaptive synaptic plasticity, glial–immune signaling, oxidative–metabolic stress, and activity-dependent gene regulation. Although conceptually separable, these domains are biologically intertwined and jointly bias circuits toward reduced resilience and unstable state transitions. Each domain has plausible links to specific neurobehavioral phenotypes, providing a mechanistic bridge between molecular alterations and system-level function [1,15,42,43].

Throughout Section 4, evidence is stratified as (i) preclinical mechanistic support (intervention or genetic manipulation) versus (ii) associative human observations (e.g., resected tissue transcriptomics, imaging correlations), which inform plausibility but do not establish causal direction.

Representative anchors used in Section 4 include IL-1R/TLR4–NF-κB and TNF-α/TNFR1 (glial–immune); mTOR and BDNF–TrkB (plasticity); Nrf2-related antioxidant signaling and mitochondrial dysfunction pathways (metabolic); and activity-dependent transcriptional/epigenetic regulators including immediate early genes (FOS, ARC, EGR1), DNMT/HDAC-dependent chromatin regulation, and epilepsy-relevant microRNAs (miR-134, miR-146a, miR-128) (gene regulation) (Box 1). These anchors are used to keep the discussion mechanistically grounded while maintaining an integrative cross-model perspective.

Box 1 **Representative molecular nodes in epileptogenesis.**

Several molecular nodes repeatedly emerge across experimental models of epileptogenesis and provide mechanistic anchors for the pathways discussed in this review.

Representative examples include:

- Activity-dependent transcription factors and immediate early genes: FOS, ARC, EGR1;
- Plasticity-related signaling pathways: BDNF–TrkB, mTOR;
- Regulators of excitation–inhibition balance: GRIN2B, GAD1/2, SLC12A5 (KCC2);
- Epigenetic modifiers: DNMT1/3A, HDAC2/4, MECP2;
- Regulatory microRNAs involved in synaptic and inflammatory modulation: miR-134, miR-146a, miR-128.

These nodes represent experimentally documented molecular regulators that link upstream epileptogenic signaling to persistent alterations in synaptic architecture, network excitability, and circuit stability.

### 4.1. Maladaptive Synaptic Plasticity and Metaplasticity

Synaptic plasticity is a central mechanism of epileptogenic remodeling. Following an initial insult, activity-dependent mechanisms that normally support learning and adaptation can become dysregulated, leading to persistent shifts in excitation–inhibition balance, altered receptor composition, and aberrant strengthening or weakening of synaptic connectivity. long-term potentiation (LTP) / long-term depression (LTD) balance may shift toward pathological operating points, while homeostatic plasticity mechanisms that stabilize firing rates can fail or overshoot. Such maladaptive plasticity not only increases excitability but also alters the rules by which circuits respond to subsequent activity patterns [44,45,46,47]. Metaplasticity—the plasticity of synaptic plasticity—provides a useful conceptual framework. Epileptogenic processes can alter thresholds for synaptic modification, rendering circuits either hyperresponsive or rigid. In cortico-hippocampal and cortico-striatal networks, such changes may impair flexible updating of action–outcome associations, response inhibition, and adaptive learning. From a behavioral perspective, these changes map onto deficits in executive control and cognitive flexibility, which can emerge early and persist across disease stages [21,35,39,44].

Maladaptive plasticity is not confined to principal excitatory neurons. Alterations in inhibitory interneuron function, synaptic targeting, and short-term plasticity can further destabilize network dynamics. Subtle inhibitory deficits may not immediately produce seizures but can reduce the robustness of information processing, thereby increasing susceptibility to stressors and perturbations. Such subthreshold network instability provides a plausible molecular–circuit substrate for trait-like neurobehavioral alterations that precede overt epilepsy [2,3,48,49].

### 4.2. Glial–Immune Signaling and Neuroinflammation

Neuroinflammatory processes are increasingly considered integral components of epileptogenesis rather than purely secondary responses to seizures. Activation of microglia and astrocytes, release of pro-inflammatory cytokines, complement signaling, and disruption of blood–brain barrier integrity can all occur early after an epileptogenic insult. These processes modulate synaptic transmission, plasticity, and neuronal excitability, often in region- and cell-type-specific ways. Crucially, inflammatory signaling can persist even in the absence of recurrent seizures, shaping long-term network function [42,50,51,52].

Glial–immune signaling exerts particularly strong influence on circuits involved in affect regulation and stress responsiveness, including limbic and prefrontal networks. Cytokine-mediated modulation of neurotransmitter systems, synaptic pruning via complement pathways, and astrocytic regulation of extracellular ion and neurotransmitter homeostasis can bias emotional processing and threat sensitivity. These mechanisms provide a biologically grounded link between inflammatory cascades and stable alterations in anxiety-like behavior, emotional regulation, and stress reactivity observed during epileptogenic remodeling [53,54,55,56].

Preclinical intervention studies targeting IL-1R/TLR4 or TNF-α signaling provide partial causal support for inflammatory contributions to network instability. In contrast, most human evidence linking inflammatory markers to affective or cognitive phenotypes remains correlational. At the circuit level, cytokine-mediated modulation of glutamatergic and GABAergic transmission can alter functional connectivity within prefrontal–amygdala and prefrontal–striatal loops. Complement-mediated synaptic pruning and astrocytic dysregulation of extracellular glutamate may further shift effective connectivity and gain control, thereby biasing salience attribution and threat-related processing.

An important feature of neuroinflammatory pathways is their bidirectional coupling with neuronal activity. Network hyperactivity can amplify inflammatory signaling, while inflammation can lower thresholds for pathological synchronization. This bidirectional coupling can sustain inflammatory tone and gradually reshape circuit function over time. Neurobehavioral phenotypes emerging in this context may therefore index persistent glial–immune engagement not captured by seizure frequency alone [42,57,58,59].

### 4.3. Oxidative and Metabolic Stress

Epileptogenesis is associated with sustained alterations in cellular metabolism and redox balance. Mitochondrial dysfunction, impaired energy utilization, and increased oxidative stress have been documented across models of acquired and genetic epilepsy. These changes can affect neuronal excitability directly, but they also influence synaptic plasticity, glial support functions, and vulnerability to subsequent insults. Metabolic stress may contribute to epileptogenic remodeling and may also be amplified by it [60,61,62,63]. Experimental modulation of metabolic pathways in preclinical models alters excitability and seizure susceptibility, supporting mechanistic involvement. However, human associations between metabolic markers and neurobehavioral phenotypes are largely indirect and require longitudinal validation.

Metabolic inefficiency reduces energetic reserve for flexible information processing, particularly in high-demand control circuits. This constraint can manifest as reduced cognitive flexibility, slowed processing speed, and motivational deficits, which may emerge during epileptogenesis independent of cumulative seizure exposure [39,64,65,66].

Importantly, metabolic pathways are modifiable and interact with inflammatory and plasticity-related mechanisms. Shifts in substrate utilization, redox signaling, and mitochondrial dynamics can influence synaptic strength and glial–neuronal coupling. This integrative role makes metabolic stress a compelling node linking molecular change to neurobehavioral output, particularly in domains such as fatigue, motivation, and executive endurance [64,66,67,68].

### 4.4. Activity-Dependent Gene Regulation and Epigenetic Remodeling

Sustained alterations in neuronal activity during epileptogenesis engage transcriptional and epigenetic mechanisms that can stabilize maladaptive network states over time. At the molecular level, these mechanisms involve defined regulatory nodes including immediate early genes (FOS, ARC, EGR1), plasticity-related signaling pathways such as BDNF–TrkB and mTOR, chromatin-modifying enzymes (DNMT1/3A, HDAC2/4), and regulatory microRNAs (e.g., miR-134, miR-146a, miR-128) that collectively modulate synaptic plasticity, receptor trafficking, and inflammatory signaling during epileptogenic remodeling. Immediate early genes, transcription factors, non-coding RNAs, and chromatin modifications contribute to long-lasting changes in receptor expression, synaptic architecture, and glial–neuronal coupling [47,69,70,71,72,73,74]. Unlike transient signaling cascades, gene regulatory and epigenomic changes may persist beyond the initial insult and thus represent candidate mechanisms for longer-term stage-dependent remodeling. Human transcriptomic evidence largely derives from cross-sectional resected tissue analyses, which precludes temporal directionality inference and requires cautious extrapolation to pre-seizure stages. Persistent gene regulatory states may underlie trait-like neurobehavioral phenotypes. Once established, altered transcriptional landscapes may bias neuronal and glial responses to subsequent activity, stress exposure, or metabolic challenge. At the behavioral level, this may manifest as relatively stable patterns of executive, affective, or motivational function. These patterns may resist short-term fluctuations yet remain modifiable under targeted molecular intervention [75,76,77,78]. Distinct initiating insults activate partially different upstream cascades. However, they frequently converge on shared transcriptional and chromatin-regulatory pathways that reshape synaptic and inflammatory gene networks [18,43,79,80]. This convergence increases the plausibility that shared molecular states may underlie cross-etiology neurobehavioral signatures. By altering ion channel expression, receptor subunit composition, and synaptic scaffolding proteins, sustained transcriptional shifts can modify intrinsic neuronal excitability and long-term circuit responsiveness, thereby stabilizing altered operating regimes across distributed networks. Importantly, most human transcriptomic evidence derives from cross-sectional resected tissue analyses, which precludes temporal directionality inference and requires cautious extrapolation to pre-seizure stages.

#### 4.4.1. Immediate Early Gene Activation (Minutes to Hours)

Acute hyperexcitability rapidly induces immediate early genes (IEGs), including FOS (c-Fos), JUN, EGR1, and ARC. These genes function as activity-dependent transcriptional regulators and are widely used as markers of neuronal activation. In experimental epilepsy models, increased c-Fos and Arc expression has been demonstrated in hippocampal and limbic circuits during early epileptogenic stages, reflecting intense synaptic remodeling pressure [47,69,70,71,72,73,74].

Although IEG expression is primarily considered an activity marker rather than a direct causal driver, Arc-dependent modulation of synaptic plasticity and AMPA receptor trafficking suggests that early transcriptional events may influence plasticity thresholds. These early transcriptional responses are best treated as enabling conditions for longer-term network reconfiguration rather than deterministic epileptogenic switches. Intervention-based modulation of selected activity-dependent transcriptional nodes in experimental models has demonstrated partial attenuation of synaptic remodeling and seizure susceptibility, providing conditional mechanistic support beyond purely descriptive activation markers.

#### 4.4.2. Plasticity-Related Transcriptional Shifts (Hours to Days)

Following immediate early responses, transcriptional changes extend to genes regulating synaptic potentiation and excitation–inhibition balance [81].

Representative pathways and nodes include:

- BDNF–TrkB signaling [82,83];
- NMDA receptor subunit composition (e.g., GRIN2B) [81];
- GAD1/GAD2 (GABA synthesis enzymes);
- SLC12A5 (KCC2 chloride transporter) [84,85];
- mTOR pathway components [86].

These molecular nodes represent experimentally documented regulatory points linking activity-dependent signaling to synaptic remodeling, excitation–inhibition balance, and network hyperexcitability during epileptogenesis.

At the circuit level, BDNF–TrkB- and mTOR-associated transcriptional shifts bias hippocampo-prefrontal plasticity rules and may manifest as reduced reversal learning efficiency and increased perseverative errors (cognitive flexibility domain). In parallel, SLC12A5 (KCC2)-linked chloride dysregulation can weaken inhibitory control in prefrontal networks, providing a mechanistic bridge to response inhibition deficits and increased interference costs in executive control tasks.

BDNF–TrkB signaling has been implicated in mossy fiber sprouting and synaptic potentiation during temporal lobe epileptogenesis [82,83], and mTOR pathway activation has been linked to structural plasticity and seizure susceptibility [86]. Downregulation of KCC2 and altered inhibitory gene expression can impair chloride homeostasis, weakening inhibitory control and destabilizing network gain.

In preclinical models, pharmacological or genetic modulation of TrkB or mTOR signaling alters seizure susceptibility and synaptic remodeling, supporting partial mechanistic causality. In contrast, human data remain largely associative, inferred from transcriptomic analyses of resected tissue or imaging correlates.

Collectively, these shifts provide a stage-anchored molecular rationale for early executive and flexibility phenotypes.

#### 4.4.3. Epigenetic Consolidation (Days to Weeks)

Longer-term stabilization of epileptogenic states involves epigenetic regulators, including:

- DNA methyltransferases (DNMT1, DNMT3A);
- Histone deacetylases (HDAC2, HDAC4);
- MECP2;
- Histone modifications such as H3K9 acetylation or H3K27 trimethylation.

Altered DNA methylation patterns and chromatin remodeling have been reported in hippocampal tissue after status epilepticus [70]. HDAC-dependent regulation modulates genes involved in synaptic transmission and inflammatory signaling, and HDAC inhibition can influence seizure thresholds and plasticity-related gene expression in experimental models, although translational interpretation remains cautious. Because DNMT/HDAC-dependent chromatin states can persist for weeks, they provide a plausible molecular substrate for the relative stability of neurobehavioral signatures across repeated assessments, particularly in executive–affective regulation domains.

Epigenetic mechanisms may stabilize transcriptional bias within limbic–prefrontal circuits and contribute to enduring alterations in emotional regulation and stress responsiveness.

#### 4.4.4. MicroRNAs and Persistent Regulatory Fine-Tuning

MicroRNAs (miRNAs) represent an additional regulatory layer in epileptogenesis.

Representative examples include:

- miR-134 (dendritic spine regulation; Henshall group);
- miR-146a (inflammatory signaling modulation);
- miR-128 (neuronal excitability regulation).

Experimental manipulation of specific miRNAs in rodent models alters seizure susceptibility and network excitability [87,88]. For example, inhibition of miR-134 reduces seizure severity in preclinical studies, and miR-146a modulates inflammatory cascades intersecting with IL-1β/TNF-α signaling [89,90].

Inflammation-linked miRNA modulation may preferentially influence limbic circuitry, with downstream effects on affective and motivational domains.

##### Integrative Temporal Layering

To clarify stage-dependent dynamics, representative patterns reported in experimental epileptogenesis are summarized below (direction and timing vary by model and region):

- Acute (minutes–hours): rapid induction of IEGs (e.g., FOS, ARC, EGR1), typically peaking within hours, often accompanied by early glial–immune signaling (e.g., IL-1β/TNF-α release, complement engagement) that may influence subsequent plasticity trajectories.
- Subacute/latent (hours–days): sustained engagement of plasticity-related signaling (e.g., BDNF–TrkB, mTOR) and shifts in excitation–inhibition homeostasis (e.g., GRIN2B, GAD1/2, SLC12A5/KCC2), aligning with emerging deficits in response inhibition and reversal/set-shifting performance.
- Consolidation (days–weeks): stabilization of transcriptional bias via chromatin and methylation regulators (e.g., DNMTs, HDACs, MECP2), providing a molecular substrate for persistent executive–affective phenotypes.
- Maintenance (weeks): miRNA-mediated fine-tuning (e.g., miR-134, miR-146a, miR-128) intersecting with dendritic spine regulation and inflammatory tone, with preferential mapping to cognitive rigidity and affective/motivational bias.

These stage-linked associations are framed as preferential and testable rather than deterministic one-to-one mappings. The layers are partially overlapping and interact bidirectionally with synaptic and inflammatory processes rather than proceeding in a strictly sequential manner [47,69,70,71,87,88,89,90,91].

Persistent transcriptional bias may contribute to:

- Reduced cognitive flexibility (plasticity threshold shifts);
- Impaired inhibitory control (chloride dysregulation, interneuron vulnerability);
- Altered threat processing (inflammation–transcription coupling);
- Motivational alterations (metabolic–transcription interactions).

Preclinical evidence supports mechanistic involvement of several pathways; however, human data linking specific transcriptional alterations to defined neurobehavioral signatures remain predominantly associative. Establishing causal direction will require longitudinal multimodal designs.

#### 4.4.5. Summary of Gene-Level Anchors Across Stages

Together, these transcriptional and epigenetic mechanisms illustrate how upstream epileptogenic signaling cascades are translated into stable molecular states that influence synaptic structure, inhibitory control, and network excitability. By anchoring the framework in specific genes and regulatory pathways, these mechanisms provide a mechanistic bridge between molecular epileptogenesis and the neurobehavioral phenotypes discussed in subsequent sections.

To enhance mechanistic specificity and clarify stage-dependent molecular anchoring, Table 1 summarizes representative genes and molecular nodes implicated in activity-dependent transcriptional and epigenetic remodeling during epileptogenesis, their approximate temporal positioning, and corresponding neurobehavioral domains most plausibly affected.

Table 1 summarizes representative molecular nodes implicated in activity-dependent transcriptional and epigenetic remodeling during epileptogenesis, their approximate temporal positioning, and preferential neurobehavioral domains. Temporal staging reflects representative experimental observations (early: minutes–hours; intermediate: hours–days; consolidation: days–weeks). Associations are preferential rather than exclusive and are supported by experimental and review data on activity-dependent gene regulation [47,69,70,71,72,73,74,92], BDNF–TrkB signaling [82,83], mTOR pathway modulation [86], chloride homeostasis [84,85], epigenetic regulation in epilepsy [70], and microRNA involvement [87,88,89,90,91].

Table 2 provides stage-linked integration of molecular nodes with vulnerable circuits and example translational behavioral readouts.

Representative molecular nodes and their temporal positioning are supported by experimental and review data on activity-dependent gene regulation [47,69,70,71,72,73,74,92], BDNF–TrkB and related plasticity signaling [82,83], mTOR pathway engagement [86], NMDA receptor and chloride homeostasis regulation [81,84,85], epigenetic remodeling in epileptogenesis [18,43,70,79,80], and microRNA involvement in network stabilization [87,88,89,90,91]. Associations are preferential rather than deterministic, and temporal staging reflects experimental observations that may vary across etiologies and models.

## 5. Neurobehavioral Phenotypes Emerging During Epileptogenic Remodeling

Neurobehavioral alterations during epileptogenesis are heterogeneous and context-dependent but cluster around specific functional network domains. These domains share two defining features: they rely on distributed cortico-limbic and cortico-striatal networks, and they depend on precisely regulated excitation–inhibition balance, synaptic plasticity, and neuromodulatory tone. As such, they are particularly sensitive to the molecular programs described in Section 3. Rather than being limited to nonspecific psychological reactions or late seizure consequences, these phenotypes can be interpreted as early functional expressions of epileptogenic network reconfiguration [3,27,32,65].

A critical distinction in this context is between transient, state-dependent fluctuations and stable, trait-like alterations. The focus here is on the latter—patterns of performance or behavior that persist across time and contexts and can be tracked longitudinally. Such trait-like phenotypes are more likely to reflect enduring changes in circuit organization and therefore have greater potential value as biomarkers of epileptogenesis [20,33,36,93].

### 5.1. Executive Control and Inhibitory Regulation

Executive control, including response inhibition, conflict monitoring, and goal-directed regulation, is consistently vulnerable during epileptogenic remodeling. These functions depend heavily on prefrontal–striatal and prefrontal–thalamic circuits that are sensitive to disruptions in inhibitory interneuron function, synaptic plasticity, and neuromodulatory balance. Even subtle impairments in these circuits can degrade the ability to suppress prepotent responses or flexibly adjust behavior in response to changing demands [93,94,95,96].

In experimental models, alterations in executive-like functions—often operationalized through tasks assessing impulse control or rule adherence—can emerge during epileptogenesis independently of seizure expression. In clinical settings, analogous deficits in inhibitory regulation and attentional control are frequently reported early in the disease course. Within this framework, such deficits may reflect early circuit instability rather than cumulative structural damage [21,39,97,98].

From a biomarker perspective, executive control measures are sensitive to prefrontal network integrity and suitable for repeated assessment, but require careful control for fatigue, medication, and mood effects [16,31,99,100]. Task paradigms with established construct validity (e.g., stop-signal, probabilistic reversal learning, effort-discounting) are preferable to global cognitive composites when mechanistic interpretability is required.

### 5.2. Cognitive Flexibility and Adaptive Learning

Cognitive flexibility—the capacity to update behavior in response to changing contingencies—is frequently affected during epileptogenic remodeling. This function depends on coordinated interactions between hippocampal, prefrontal, and striatal networks and is tightly coupled to synaptic plasticity mechanisms. Alterations in plasticity thresholds and metaplastic regulation, as described in the section “Why Neurobehavioral Signatures Can Be Informative Biomarkers”, can therefore manifest as rigidity in learning strategies and impaired adaptation to novel or shifting task demands [44,45,46,47].

In animal models, deficits in reversal learning, strategy switching, and pattern separation have been reported during epileptogenesis, sometimes preceding recurrent seizures. In humans, comparable difficulties with adapting to new rules or integrating feedback are observed across epilepsy syndromes, often early and persistently. These findings support the interpretation that impaired flexibility reflects network-level consequences of altered plasticity rather than generalized cognitive decline [5,6,97,101]. As candidate biomarkers, measures of cognitive flexibility are particularly informative because they probe dynamic updating rather than static performance. Longitudinal changes in flexibility measures may index the trajectory of network remodeling and the impact of interventions targeting adaptive plasticity [16,29,36,102].

### 5.3. Emotional Regulation and Threat Processing

Alterations in emotional regulation and threat processing are consistently reported during epileptogenesis. These changes are closely linked to limbic and paralimbic circuits that are highly sensitive to neuroinflammatory signaling, neuromodulatory shifts, and synaptic remodeling. Heightened threat sensitivity, altered stress reactivity, and impaired extinction of aversive responses can emerge as stable traits rather than transient state-dependent emotional responses [50,93,100,103]. Within this framework, threat-processing alterations are distinguished from generalized anxiety or formal psychiatric diagnoses. We focus on task-defined biases (e.g., extinction rate, threat-bias indices, avoidance learning dynamics) rather than symptom-based anxiety constructs.

Importantly, such phenotypes should not be conflated with formal psychiatric diagnoses. Within this framework, threat-processing alterations are distinguished from formal psychiatric diagnoses and are defined using task-based metrics (e.g., extinction rate, threat-bias indices, avoidance learning dynamics). These phenotypes are interpreted as functional readouts of altered amygdala–hippocampal–prefrontal circuit dynamics [55,95,96,98].

Emotional regulation measures pose specific challenges as biomarkers, given their susceptibility to contextual influences and subjective reporting. Nevertheless, when anchored to objective tasks or physiological correlates and assessed longitudinally, they may provide valuable information about disease activity in circuits that are central to epileptogenic progression [33,34,93,95].

### 5.4. Motivational and Social Behavior

Motivational and social behaviors constitute an additional domain affected by epileptogenic remodeling. These functions depend on distributed networks involving prefrontal cortex, limbic structures, and mesolimbic systems that integrate cognitive, emotional, and reward-related information. Their emergence during epileptogenesis suggests broader network reconfiguration rather than purely situational or psychosocial effects [22,23,38,98]. Importantly, reduced reward sensitivity or effort allocation should not be equated with social withdrawal per se. Effort-based decision-making, progressive ratio performance, and reward discounting paradigms capture motivational valuation processes that may dissociate from social engagement measures.

In both experimental and clinical contexts, changes in motivation and social interaction are often underappreciated because they may not directly relate to seizure control. However, their emergence during epileptogenesis suggests that they reflect broader network reconfiguration rather than secondary psychosocial or situational effects. Interpreted as neurobehavioral signatures, such changes may index dysfunction in circuits that are also critical for adaptive decision-making and long-term outcome [22,23,38,98].

From a translational standpoint, motivational and social measures may complement executive and affective domains by capturing aspects of network function that are less tied to overt task performance. When integrated with molecular and electrophysiological markers, motivational and social phenotypes may enhance multidimensional characterization of epileptogenic progression [18,32,40,104,105].

## 6. Integrative Mapping: Aligning Molecular Cascades with Neurobehavioral Signatures

This section aligns molecular epileptogenic domains with vulnerable circuits and neurobehavioral readouts, using Table 3 as a structured reference. Rather than assuming one-to-one correspondence between molecular pathways and behavioral outcomes, the mapping identifies preferential associations between molecular cascades, circuit vulnerabilities, and neurobehavioral domains [13,15,32,33,34].

Across Section 3 and Section 4, four recurring molecular domains—maladaptive synaptic plasticity, glial–immune signaling, oxidative–metabolic stress, and activity-dependent gene regulation—were shown to align with executive control, cognitive flexibility, emotional regulation, and motivational–social behavior. Table 3 summarizes these relationships to support mechanistic interpretation and biomarker-oriented study design.

This mapping is intended as a structured analytical scaffold for testable longitudinal and interventional study designs.

### 6.1. Maladaptive Synaptic Plasticity: Executive Control and Cognitive Flexibility

Maladaptive synaptic plasticity and altered metaplastic thresholds (Section 3) preferentially affect circuits supporting executive control and cognitive flexibility. Prefrontal–striatal and prefrontal–hippocampal networks rely on finely tuned plasticity rules to support response inhibition, rule updating, and adaptive learning. When plasticity rules are biased—through altered receptor trafficking, impaired inhibitory regulation, or failed homeostatic compensation—circuits may become excessively rigid or pathologically labile [21,35,39,44]. From a neurobehavioral standpoint, this manifests as reduced inhibitory control, impaired set-shifting, and diminished adaptability to changing contingencies. These phenotypes may index early disruption of plasticity-dependent network functions and can be operationalized through executive and flexibility-related behavioral readouts (Table 1) [5,6,28,97].

### 6.2. Glial–Immune Signaling: Emotional Regulation and Threat Processing

Glial–immune signaling and neuroinflammatory cascades (Section 3) align with alterations in emotional regulation and threat processing. Limbic circuits, including amygdala–hippocampal–prefrontal pathways, are particularly sensitive to cytokine signaling, complement-mediated synaptic remodeling, and astrocytic modulation of extracellular homeostasis. Persistent inflammatory engagement can therefore bias these circuits toward heightened salience attribution, altered stress reactivity, and reduced regulatory control [50,55,56,103].

Neurobehavioral signatures in this domain—such as stable elevations in threat sensitivity or altered extinction learning—may reflect sustained glial–immune engagement. Emotional regulation measures can therefore complement molecular and imaging markers that capture inflammatory activity more directly [51,53,54,100].

### 6.3. Oxidative–Metabolic Stress: Motivation and Executive Endurance

Oxidative and metabolic stress preferentially constrain circuits requiring sustained energetic support, including those underlying motivation, goal-directed behavior, and executive endurance. Mitochondrial dysfunction and redox imbalance reduce energetic reserve for flexible information processing, biasing networks toward conservation rather than adaptation [62,63,64,65]. Fast-spiking inhibitory interneurons, which have high metabolic demand, may be particularly vulnerable, weakening inhibitory buffering capacity and destabilizing network gain.

Behaviorally, this may manifest as reduced motivation, diminished reward sensitivity, or early cognitive fatigue. In Table 1, metabolic markers are aligned with motivational and endurance-related measures, illustrating how energetic constraints translate into stable behavioral change during epileptogenesis [67,68,94,104].

### 6.4. Activity-Dependent Gene Regulation: Trait-like Stability of Neurobehavioral Phenotypes

Activity-dependent transcriptional and epigenetic remodeling provides a mechanistic substrate for the trait-like stability of neurobehavioral signatures across domains. Persistent changes in gene expression and chromatin state can stabilize altered synaptic and glial phenotypes, biasing circuit responses across time and contexts [70,77,105,106,107].

Diverse epileptogenic insults may engage distinct upstream pathways yet converge on shared gene regulatory states that support similar neurobehavioral phenotypes [18,43,75,76]. This convergence is relevant for biomarker development, as trait-like behavioral signatures may index common downstream disease mechanisms across etiologies.

### 6.5. Toward an Operational Integrative Framework

Table 3 operationalizes this alignment by linking molecular domains to preferentially associated neurobehavioral phenotypes and vulnerable circuits. This structured mapping supports mechanistic hypothesis generation and integration of behavioral endpoints into multimodal biomarker strategies. Validation will require longitudinal and interventional studies.

Explicit alignment of molecular cascades with neurobehavioral signatures facilitates identification of mechanistically grounded behavioral markers and supports integration of behavioral endpoints into multimodal biomarker strategies. Validation will require longitudinal and interventional studies.

These advances set the stage for the methodological considerations addressed in the next section, in which criteria for biomarker validity are examined in detail [18,40,105,108,109].

## 7. Translational Biomarker Validity: Methodological Requirements

To function as translational biomarkers, neurobehavioral signatures must meet predefined methodological criteria, including mechanistic interpretability, temporal anchoring, and reproducibility across models and contexts. This section focuses on three core requirements—specificity, temporal anchoring, and cross-model consistency—and summarizes practical design strategies to address common sources of bias and confounding [9,10,11,12].

### 7.1. Specificity and Control of Confounding

Specificity is a primary challenge for neurobehavioral biomarkers in epilepsy. Cognitive and affective measures are sensitive to multiple influences, including seizure burden, antiseizure medication effects, sleep disruption, pain, stress, and psychosocial context. Without explicit control of these factors, neurobehavioral changes may be misattributed to epileptogenic mechanisms. Study designs should either minimize confounding or model its contribution explicitly [29,31,38,100].

Several methodological strategies can enhance specificity. First, inclusion criteria should restrict recent seizure exposure and medication changes when the goal is to probe pre-seizure or early epileptogenic stages. Second, parallel measurement of confounders—such as sleep quality, mood, and fatigue—should be incorporated as covariates rather than treated as exclusionary noise. Third, inclusion of negative control domains—functions not expected to be affected by the molecular pathways under investigation—can help distinguish domain-specific effects from generalized impairment [9,16,36,102].

Specificity requires demonstrable linkage to epileptogenic mechanisms. Neurobehavioral measures that covary with molecular or electrophysiological markers of disease activity, but not with unrelated stressors, provide stronger evidence of biomarker relevance [10,11,12,17].

### 7.2. Temporal Anchoring and Longitudinal Design

Temporal anchoring means that biomarker changes align with defined stages of epileptogenesis. For neurobehavioral signatures, this requires longitudinal designs that separate early progressive changes from late consequences of established epilepsy [1,19,36,101].

Temporal anchoring requires that biomarker changes align with defined stages of epileptogenesis and be dissociable from late consequences of established epilepsy [1,19,36,101].

A critical methodological consideration is the stability of neurobehavioral measures across repeated testing. Biomarkers intended to track progression must demonstrate acceptable test–retest reliability and sensitivity to gradual change rather than acute fluctuation. Task selection should prioritize longitudinal robustness over maximal cross-sectional discrimination [16,30,36,110].

Interictal epileptiform discharges can exert measurable, state- and task-dependent effects on cognition (e.g., language, attention, executive performance). Neurobehavioral analyses should therefore account for interictal activity burden and its timing relative to testing to avoid misattributing transient electrophysiological instability to epileptogenic progression [111,112,113,114,115,116,117,118,119,120].

### 7.3. Cross-Model Consistency and Translational Triangulation

Cross-model consistency strengthens translational validity by reducing reliance on model- or cohort-specific effects [1,86,121,122].

Translational triangulation involves aligning neurobehavioral phenotypes with molecular and electrophysiological markers across levels of analysis. For example, a behavioral alteration that covaries with inflammatory markers in animal models and with imaging or peripheral biomarkers in humans provides stronger mechanistic support than behavioral data alone. Triangulation reduces reliance on a single modality and improves mechanistic interpretation [32,33,40,105].

Perfect cross-model equivalence is neither expected nor required; the key objective is domain-level convergence that can be mechanistically linked despite differences in experimental implementation [11,18,108,123]. Although exact task equivalence between rodent and human paradigms is not assumed, directional convergence at the domain level (e.g., reversal learning deficits, extinction learning alterations, effort allocation bias) supports construct-level alignment rather than one-to-one task translation.

### 7.4. Measurement Selection and Analytical Considerations

Selection of neurobehavioral measures has direct implications for biomarker validity. Tasks should be selected based on mechanistic relevance to vulnerable circuits rather than on convenience or clinical familiarity. Quantitative metrics capturing variability, error patterns, or learning dynamics may be more informative than summary scores alone [16,21,35,93]. Mixed-effects models and within-subject designs help separate trait-like change from state-dependent variability. Where feasible, preregistration of primary behavioral endpoints and analytical plans can reduce interpretive bias and improve reproducibility [11,16,36,102].

Autonomic and psychophysiological signals (e.g., electrodermal activity) may provide complementary readouts of state-dependent network instability and, in some contexts, correlate with seizure susceptibility. When integrated with behavioral and electrophysiological measures, such signals can serve as intermediate phenotypes linking network excitability to observable behavior [124,125,126,127,128,129,130,131,132,133].

### 7.5. Implications for Biomarker Qualification

When these requirements are met, neurobehavioral signatures may serve as biomarkers for defined translational use cases (e.g., disease staging, intervention timing, or pharmacodynamic readouts). Intended use should be specified explicitly, as the same measure may function as a progression biomarker in one setting and as a pharmacodynamic marker in another [9,10,11,12]. Neurobehavioral signatures are most informative when embedded in multimodal strategies. Such integration constrains interpretation and improves translational relevance. The following section builds on this foundation to discuss how such biomarkers can inform the development and evaluation of anti-epileptogenic strategies.

Multimodal integration may incorporate peripheral candidates (e.g., IL-1β, TNF-α; oxidative–metabolic indices) and imaging correlates (e.g., functional connectivity; PET-based neuroinflammation/microglial markers) as context-sensitive signals within predefined intended-use frameworks rather than standalone diagnostic indicators. For pharmacodynamic applications, parallel shifts in domain-specific behavioral metrics together with electrophysiological or network-level markers (e.g., EEG-derived instability indices) can support early assessment of target engagement in disease-modifying studies.

**Figure 3:**
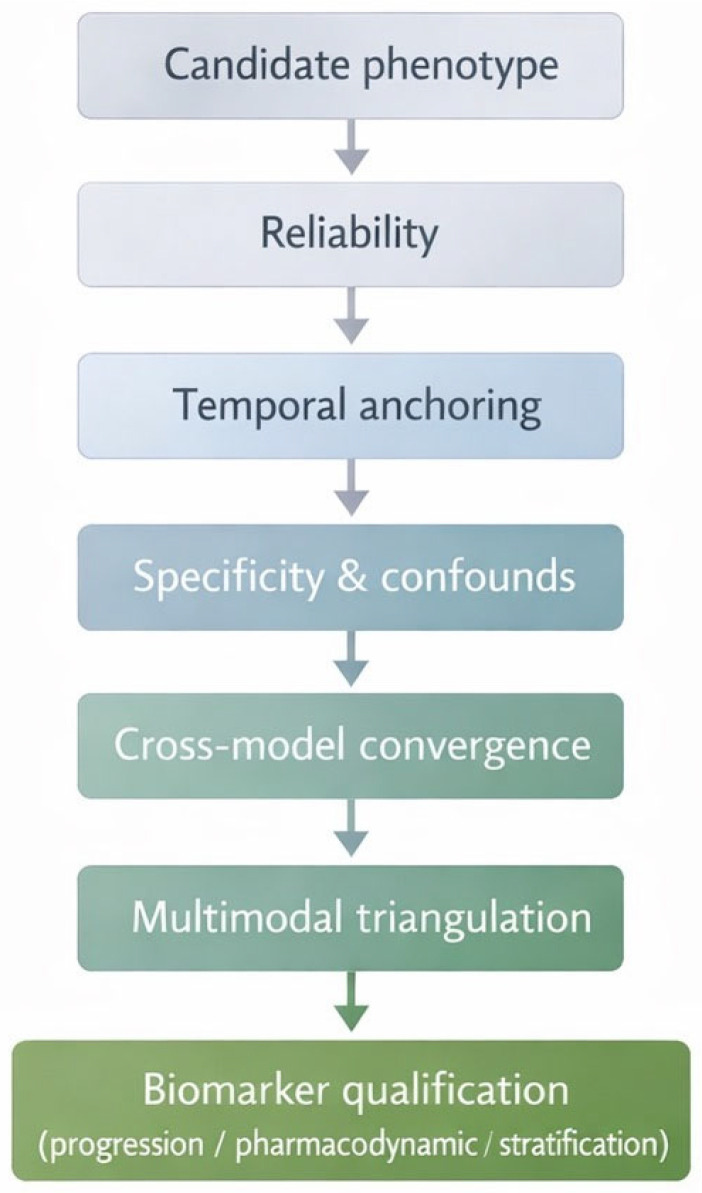
Biomarker qualification workflow for neurobehavioral signatures.

Table 4 summarizes representative neurobehavioral readouts, key confounders, and minimal controls for rigorous interpretation. Figure 3 outlines a stepwise workflow for biomarker qualification from phenotype identification to intended-use definition.

To formalize these requirements, Figure 3 summarizes a stepwise biomarker qualification workflow for neurobehavioral signatures, from initial phenotype identification to defined translational use.

Schematic stepwise process for evaluating neurobehavioral phenotypes as candidate biomarkers of epileptogenesis, progressing from phenotype identification through reliability testing, temporal anchoring, specificity and confounding control, cross-model convergence, and multimodal triangulation toward defined translational use (e.g., progression, pharmacodynamic response, or stratification). Peripheral, imaging, and electrophysiological markers are integrated as contextual modifiers within predefined intended-use frameworks rather than as standalone diagnostic indicators.

## 8. Implications for Anti-Epileptogenic Strategies

**Figure 4:**
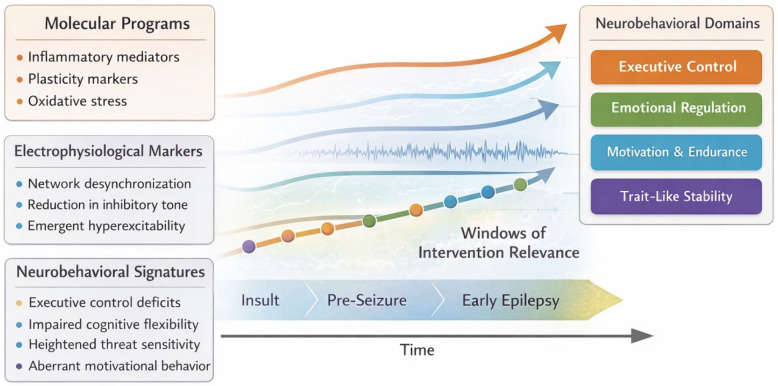
Neurobehavioral signatures as integrative readouts across stages of epileptogenesis.

The temporal positioning of neurobehavioral signatures across stages of epileptogenes and their potential relevance for intervention timing are illustrated in Figure 4.

Schematic representation of epileptogenesis from initial insult through pre-seizure phases to early epilepsy. Molecular, electrophysiological, and neurobehavioral layers are depicted as partially overlapping processes evolving over time. Shaded regions indicate periods of potential relevance for disease-modifying interventions.

An additional translational implication relates to multiday seizure cycles and rhythmic fluctuations in seizure risk. Longitudinal analyses suggest that epileptogenic processes unfold against a dynamically modulated background rather than a stationary baseline. Incorporating cycle-aware modeling into intervention studies may improve outcome sensitivity and timing precision, particularly in pre-seizure or early-stage contexts [115,134,135,136,137,138,139,140,141,142,143,144,145,146,147].

Neurobehavioral signatures may inform when and how disease-modifying interventions are evaluated. Although molecular targets have been identified, most current therapies remain primarily symptomatic and do not reliably alter epileptogenic progression once network remodeling is established. A major limitation is the scarcity of markers indexing progression prior to, or independent of, recurrent seizures [4,19,40,41].

In this framework, neurobehavioral signatures are treated as functional indicators of network vulnerability rather than therapeutic targets. Their emergence or stabilization may signal windows during which interventions are more likely to influence disease trajectory. Longitudinal phenotyping can therefore assist in identifying intervention-relevant timepoints [15,19,41,67].

Seizure occurrence is discontinuous and may be delayed relative to underlying network remodeling. In contrast, neurobehavioral measures provide repeatable functional readouts that can be tracked over time. When interpreted alongside molecular and electrophysiological markers, such measures may support early assessment of mechanism engagement, even in the absence of immediate seizure effects [19,29,37,40].

Linking domain-specific neurobehavioral changes to modifiable molecular nodes may inform target prioritization. Pathways preferentially aligned with executive, flexibility, inflammatory, or metabolic domains can be evaluated for their impact on corresponding behavioral readouts. Behavioral changes may precede detectable shifts in seizure thresholds; however, normalization of neurobehavioral phenotypes alone does not demonstrate anti-epileptogenic efficacy [50,67,68,148]. Concordant changes across neurobehavioral, molecular, and electrophysiological markers strengthen inference regarding disease-modifying engagement and underscore the importance of multimodal study designs [11,38,40,105].

Neurobehavioral signatures may also support stratification in anti-epileptogenic research. Heterogeneity in epileptogenic pathways suggests stage- and domain-specific vulnerability profiles. Alignment of neurobehavioral patterns with underlying molecular and circuit alterations may assist in subgroup identification and intervention timing [15,25,102,149].

Integration of neurobehavioral signatures with molecular and electrophysiological approaches provides temporally sensitive indicators of network state and may refine preclinical screening and clinical trial design in studies targeting epileptogenesis rather than seizure suppression alone.

## 9. Limitations and Future Directions

Several limitations inherent to the proposed framework should be acknowledged. First, neurobehavioral signatures are intrinsically multicausal and highly sensitive to contextual influences, including seizure burden, pharmacological treatment, sleep disruption, and psychosocial factors. Although strategies for confounding control are discussed above, complete dissociation of epileptogenic mechanisms from these influences is rarely achievable in practice. Accordingly, neurobehavioral measures should be interpreted within multimodal frameworks rather than in isolation. Second, heterogeneity across epileptogenic etiologies and experimental models poses a challenge for generalization. Molecular cascades and network trajectories may differ substantially between post-injury, genetic, and neurodevelopmental forms of epilepsy. As a result, neurobehavioral signatures identified in one context may not readily translate to another. Future research should therefore emphasize convergence across models and etiologies rather than universality, focusing on shared downstream mechanisms and domain-level vulnerabilities rather than identical phenotypic expressions.

In addition, sex as a biological variable remains underreported in many experimental and clinical studies of epileptogenesis, limiting assessment of potential sex-dependent trajectories. Future longitudinal designs should incorporate sex-stratified analyses where feasible, particularly for inflammatory engagement, plasticity dynamics, and neurobehavioral trajectories.

Third, limited temporal resolution remains a major constraint. Precise temporal anchoring of neurobehavioral changes to specific stages of epileptogenesis requires longitudinal designs that are both resource-intensive and methodologically demanding. In clinical settings, uncertainty regarding the true onset of epileptogenesis further complicates interpretation. Addressing this limitation will require prospective studies in high-risk cohorts, alongside improved alignment between experimental timelines and clinical disease trajectories.

Fourth, measurement-related challenges warrant careful consideration. Many commonly used cognitive and affective assessments were not designed for repeated longitudinal application and may be affected by practice effects, ceiling effects, or limited sensitivity to gradual change. Future work should prioritize the development and validation of tasks optimized for longitudinal tracking, with explicit attention to reliability, sensitivity, and mechanistic relevance.

A recurring methodological limitation in clinical studies is reliance on patient-reported seizure counts, diaries, and retrospective symptom assessments. While indispensable in clinical practice, self-reported measures are vulnerable to recall bias, unrecognized seizures, and variability in reporting accuracy. These limitations are particularly relevant when neurobehavioral outcomes are correlated with seizure burden or disease stage, as measurement noise may obscure true associations or inflate spurious ones [150,151,152,153,154,155,156].

Future work should prioritize multimodal longitudinal designs integrating neurobehavioral phenotyping with molecular, electrophysiological, and imaging markers to test the proposed mappings across contexts. Standardization of phenotyping batteries and analytical approaches would improve comparability across studies. Incorporating neurobehavioral endpoints into early-phase intervention studies as exploratory or mechanistic readouts may provide information on target engagement before seizure-based outcomes become informative.

Progress may be supported by objective digital monitoring tools, including wearable seizure detection systems, multimodal sensing frameworks, and structured self-management platforms. Such approaches can reduce reliance on subjective reporting, enable continuous within-subject assessment, and improve contextualization of neurobehavioral trajectories. Standardized quality-of-life and seizure severity instruments embedded within digital ecosystems may further support clinically meaningful, patient-centered endpoints in anti-epileptogenic trials [157,158,159,160,161,162,163,164].

## 10. Conclusions

Epileptogenesis is a progressive, multiscale disease process that unfolds long before seizures emerge as the dominant clinical manifestation. This review argues that stable neurobehavioral signatures—when rigorously defined, temporally anchored, and mechanistically integrated—may serve as informative system-level readouts of epileptogenic network remodeling. When appropriately controlled and triangulated, such phenotypes may reflect molecular and circuit-level processes contributing to seizure generation rather than nonspecific comorbidities alone.

By aligning molecular cascades with vulnerable neurobehavioral domains under explicit biomarker-validity criteria, this framework extends translational evaluation beyond seizure occurrence alone. Integrating neurobehavioral signatures with molecular and electrophysiological measures may support earlier assessment of disease activity, improved stratification, and more informative evaluation of candidate anti-epileptogenic strategies. Conceptualizing epileptogenesis through this integrative lens can inform study design for disease-modifying interventions across pre-seizure and early-stage contexts.

## Figures and Tables

**Table 1:** Gene-Level Anchoring of Activity-Dependent Remodeling Across Stages of Epileptogenesis.

Molecular Node/Gene	Functional Category	Approximate Temporal Stage	Mechanistic Role in Epileptogenesis	Preferential Neurobehavioral Domain
FOS (c-Fos)	Immediate early gene	Minutes–Hours	Activity marker; initiates transcriptional cascades	State-dependent activation; early network instability
ARC	Synaptic plasticity regulator	Minutes–Hours	AMPA receptor trafficking; synaptic scaling	Cognitive flexibility; adaptive updating
EGR1	Transcription factor	Minutes–Hours	Plasticity-related transcriptional control	Executive control modulation
BDNF	Neurotrophic factor	Hours–Days	Mossy fiber sprouting; synaptic potentiation	Cognitive flexibility; learning adaptation
NTRK2 (TrkB)	Receptor tyrosine kinase	Hours–Days	BDNF signaling; structural plasticity	Executive and hippocampal-dependent learning
MTOR	Kinase signaling hub	Hours–Days	Protein synthesis; dendritic growth; hyperexcitability	Executive endurance; network rigidity
GRIN2B	NMDA receptor subunit	Hours–Days	Excitatory synaptic remodeling	Flexibility; prefrontal–hippocampal integration
GAD1/GAD2	GABA synthesis enzymes	Subacute	Inhibitory tone regulation	Inhibitory control; executive stability
SLC12A5 (KCC2)	Chloride transporter	Subacute	E/I balance; interneuron function	Inhibitory control; threat regulation
DNMT1/DNMT3A	DNA methyltransferases	Days–Weeks	Stable transcriptional repression	Trait stabilization across domains
HDAC2/HDAC4	Histone deacetylases	Days–Weeks	Chromatin remodeling; plasticity modulation	Persistent executive/affective bias
MECP2	Chromatin regulator	Days–Weeks	Long-term gene expression control	Cross-domain stability
miR-134	microRNA	Weeks	Dendritic spine regulation	Cognitive rigidity
miR-146a	microRNA	Weeks	Inflammatory modulation	Emotional regulation bias
miR-128	microRNA	Weeks	Neuronal excitability tuning	Motivational–executive interaction

**Table 2:** Stage-Linked Integration of Molecular Nodes and Neurobehavioral Readouts.

Epileptogenesis Stage	Representative Molecular Nodes	Vulnerable Circuit Bias	Preferential Neurobehavioral Domain	Example Translational Readouts
Acute (minutes–hours)	FOS, ARC, EGR1	Hippocampal–limbic activation instability	State-dependent executive fluctuation	Response inhibition variability; task-switch cost instability
Subacute/Latent (hours–days)	BDNF–TrkB, mTOR, GRIN2B, SLC12A5	Prefrontal–hippocampal plasticity bias	Cognitive flexibility	Reversal learning errors; perseveration index; probabilistic updating deficits
Consolidation (days–weeks)	DNMT1/3A, HDAC2/4, MECP2	Prefrontal–limbic transcriptional stabilization	Executive–affective trait stabilization	Reduced intra-individual variability; persistent interference scores
Maintenance (weeks)	miR-134, miR-146a, miR-128	Limbic–striatal modulation	Emotional regulation; motivational bias	Threat-bias indices; extinction rate shifts; effort discounting slopes

**Table 3:** Molecular domains of epileptogenesis and their preferential alignment with neurobehavioral phenotypes.

Molecular Domain	Representative Mechanisms	Vulnerable Circuits	Preferential Neurobehavioral Domain	Translational Interpretation
Maladaptive synaptic plasticity	Altered LTP/LTD thresholds; impaired metaplasticity; inhibitory synapse remodeling	Prefrontal–striatal; prefrontal–hippocampal	Executive control; cognitive flexibility	Early functional marker of plasticity bias and reduced adaptive control
Glial–immune signaling	Cytokine release; complement-mediated synaptic remodeling; astrocytic homeostatic dysfunction	Limbic; prefrontal–limbic	Emotional regulation; threat processing	Proxy of persistent inflammatory engagement at the circuit level
Oxidative–metabolic stress	Mitochondrial dysfunction; redox imbalance; impaired energy utilization	Cortico-striatal; mesolimbic	Motivation; executive endurance	Indicator of energetic constraints limiting network flexibility
Activity-dependent gene regulation	Transcriptional persistence; epigenetic remodeling; long-term gene expression bias	Distributed, multi-network	Trait-like stability across behavioral domains	Convergence marker reflecting sustained network reprogramming across etiologies

Note: Alignments indicate preferential, not exclusive, relationships between molecular domains and neurobehavioral phenotypes.

**Table 4:** Proposed neurobehavioral biomarker panel: domains, readouts, confounders, and recommended controls.

Neurobehavioral Domain	Example Readouts/Tasks (Preclinical/Clinical)	Key Confounders	Minimal Controls	Intended Biomarker Use
Executive control	Set-shifting tasks, reversal learning, response inhibition paradigms/Trail Making Test (B–A), Stroop interference, Go/No-Go	Antiseizure medications (sedation, cognitive slowing); sleep deprivation; depression; fatigue	Current ASM regimen and dose; sleep duration/quality; depression screening; baseline processing speed	Disease progression; stratification
Cognitive flexibility	Attentional set-shifting, probabilistic learning/Wisconsin Card Sorting Test, task-switching paradigms	Anxiety level; test–retest learning effects; education	Anxiety scales; task counterbalancing; education level	Disease progression
Emotional regulation/threat processing	Fear conditioning/extinction, avoidance behavior/threat bias tasks, affective reactivity scales	Mood disorders; stress exposure; psychotropic co-medication	Depression/anxiety screening; recent stress assessment; medication inventory	Stratification; pharmacodynamic
Motivation and endurance	Effort-based decision-making, progressive ratio tasks/fatigue scales, effort discounting paradigms	Fatigue; pain; sleep disruption; somatic comorbidities	Fatigue and pain scales; sleep assessment; physical activity level	Disease progression; pharmacodynamic
Trait-like behavioral stability	Longitudinal behavioral consistency across tasks/intra-individual variability across repeated assessments	Practice effects; life events; aging	Repeated baseline measurements; age-matched norms; life-event tracking	Disease progression; stratification

Note: Neurobehavioral domains are shown with representative, not exhaustive, tasks. Intended biomarker use refers to exploratory roles within multimodal biomarker strategies rather than standalone diagnostic or predictive markers.

## Data Availability

No new data were created or analyzed in this study. Data sharing is not applicable to this article.

## Supplementary Materials

The following supporting information can be downloaded at: https://www.mdpi.com/article/10.3390/ijms27052511/s1.
