# Supplementary material for: Neurobehavioral Signatures of Epileptogenesis: Molecular Programs, Trait-like Phenotypes, and Translational Biomarkers Beyond Seizures
Source: Int J Mol Sci. 2026 Mar 9;27(5):2511. doi: 10.3390/ijms27052511 (PMC12985557; doi:10.3390/ijms27052511)
Supplement: Supplementary file 1 [file ijms-27-02511-s001.zip › ijms-4192059-supplementary.pdf]

International Journal of *Molecular Sciences*

MDPI

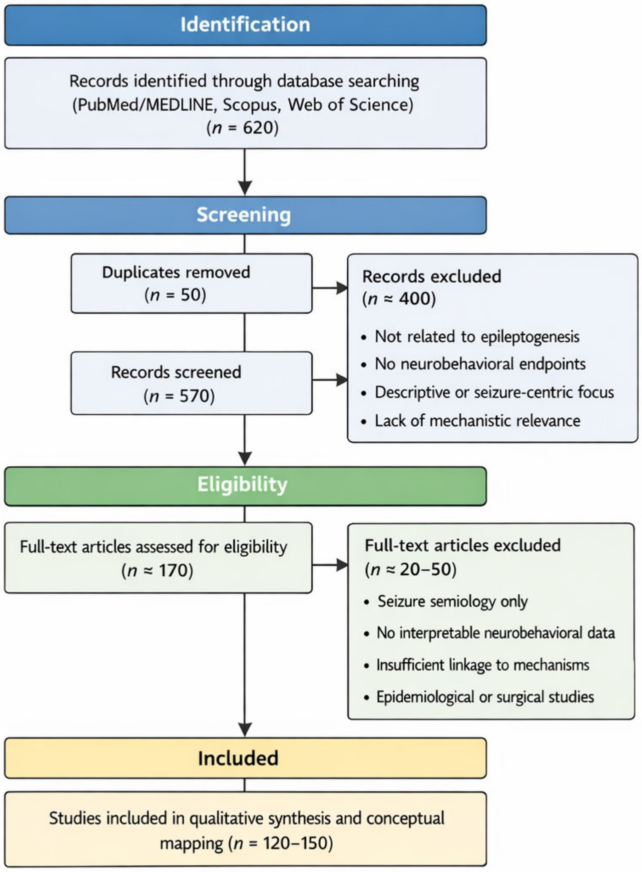

Figure S1. PRISMA-style flow diagram of study selection.

Table S1. Representative search strings used for literature retrieval across databases.

**Database**	**Search Strategy**
PubMed / MEDLINE	(epileptogenesis OR antiepileptogenic OR "latent period" OR "post-injury epilepsy") AND (cognition OR "executive function" OR "cognitive flexibility" OR anxiety OR "affect regulation" OR motivation OR "social behavior" OR "behavioral phenotype") AND ("synaptic plasticity" OR neuroinflammation OR "glial signaling" OR "oxidative stress" OR metabolism OR "gene regulation" OR epigenetic*)
Scopus	TITLE-ABS-KEY (epileptogenesis OR antiepileptogenic OR "disease modification") AND TITLE-ABS-KEY (cognition OR "executive control" OR "cognitive flexibility" OR emotional OR motivational OR social) AND TITLE-ABS-KEY ("synaptic plasticity" OR neuroinflammation OR glial OR metabolic OR transcription* OR epigenetic*)
Web of Science	TS=(epileptogenesis OR antiepileptogenic OR "latent period") AND TS=(cognition OR executive OR flexibility OR affect* OR motivation OR social)

	AND TS=("synaptic plasticity" OR neuroinflammation OR glial OR oxidative OR metabolic OR gene regulation)

Table S2. Extracted domains and conceptual framework used for integrative qualitative synthesis.

Category	Extracted Variables
Study type	Animal models; human observational or clinical studies
Epileptogenesis context	Etiology or experimental model; pre-seizure phase; early epilepsy
Neurobehavioral domain	Executive control; cognitive flexibility; emotional regulation / threat processing; motivational–social behavior
Molecular / cellular domain	Maladaptive synaptic plasticity; glial–immune signaling; oxidative–metabolic stress; activity-dependent gene regulation and epigenetic remodeling
Circuit level	Prefrontal–striatal; prefrontal–hippocampal; limbic; mesolimbic; distributed networks
Mechanistic linkage	Molecular markers; circuit-level alterations; electrophysiological correlates; imaging findings; biomarker associations
Translational relevance	Disease progression; stratification; pharmacodynamic or mechanistic readouts

This conceptual mapping framework was used to organize evidence across studies and to support the integrative alignment between molecular epileptogenic programs and neurobehavioral signatures presented in the main text.

Disclaimer/Publisher's Note: The statements, opinions and data contained in all publications are solely those of the individual author(s) and contributor(s) and not of MDPI and/or the editor(s). MDPI and/or the editor(s) disclaim responsibility for any injury to people or property resulting from any ideas, methods, instructions or products referred to in the content.
